# Occupational stress and associated factors among health care professionals in Ethiopia: a systematic review and meta-analysis

**DOI:** 10.1186/s12889-021-10579-1

**Published:** 2021-03-19

**Authors:** Bekahegn Girma, Jemberu Nigussie, Alemayehu Molla, Moges Mareg

**Affiliations:** 1 Department of Nursing, College of Medicine and Health Science, Dilla University, Dilla, Ethiopia; 2 Department of Psychiatry, College of Medicine and Health Science, Dilla University, Dilla, Ethiopia; 3 Department of Reproductive Health, College of Medicine and Health Science, School of Public Health, Dilla University, Dilla, Ethiopia

**Keywords:** Occupational stress, Health care professionals, Ethiopia, Meta-analysis

## Abstract

**Background:**

Occupational stress is a global health problem which affects employed personals especially health professionals. The burden of stress is not limited at individual level, but also affects the organizations productivity, the quality of care and country in large. In Ethiopia, little concern is given to this problem and individual studies conducted among health care professionals also showed inconsistent result. Therefore, the aim of this study was to assess the pooled prevalence of occupational stress and its associated factors among health care professionals in Ethiopia.

**Methods:**

Articles were searched from PubMed, Hinari, PsychInfo, Science direct databases, Google and Google scholar. A total of 10 studies were included in this review and meta-analysis. We used a standardized format for data extraction and STATA software version 13 for analysis. A random effect meta-analysis model was used to determine the pooled prevalence of occupational stress and I^2^ was used to check heterogeneity. Begg’s and Egger’s tests were conducted to detect publication bias. Furthermore, sensitivity and subgroup analysis was also conducted. Association was expressed by pooled odd ratio with corresponding 95% CI.

**Results:**

The pooled prevalence of occupational stress was 52.5 [95% CI: (47.03, 57.96)]. The heterogeneity test was I^2^ = 89.1% & *P* < 0.001. The result of the publication bias detection (Begg’s and Egger’s) tests were *p* = 0.283 and *p* = 0.369 respectively. Female sex was identified as a significant predictor for occupational stress with a pooled effect of 3.75 [95% CI: (2.58, 5.45)].

**Conclusions:**

Above half of health care professionals had occupational stress. Being female was significantly associated factor in this review and meta-analysis. Therefore, introduction of policies supporting health care professionals well-being at work in Ethiopia are advisable.

## Background

According to the National Institute of Occupational Safety and Health (NIOSH), occupational stress is defined as “harmful physical and emotional responses that occur when the requirements of the job do not match the capabilities, resources and needs of the worker” [1]. Stress is a response to stressors and can be positive or negative in its nature [2, 3].

Different findings showed that individuals and organizations are largely affected by occupational stress. Despite, it affects all employed professionals; the burden is too high among health care providers [4–6]. Occupational stress is a major reason for work related delay, absenteeism, hypertension, musculoskeletal disorders, cardiovascular disorders and substance use [7–11]. Moreover, it is also a major cause of mental disturbance, injuries and staff turnover [8, 12, 13]. It also reduces organizational commitment, job satisfaction, quality of care and organizational productivity [14–20]. Occupational stress is a second work related problem next to low back pain [21].

In the globe, a minimum of 3 million employees frontage grave occupational stress problems and 28% of employees in European Union are affected by occupational stress [21]. It is also responsible for 50 to 60% of losses in working days [22]. In 2019, about 83% of workers suffer from occupational stress in United States (US) and it also caused 120,000 deaths [23]. A finding in systematic review and meta-analysis showed that occupational stress is responsible for an estimated cost of $221.13 million to $187 billion; 70–90% productivity related losses [24].

The magnitude of occupational stress among health care professionals ranged from 27 to 87.4% [9, 25–29]. Studies conducted in Ethiopia among health care professionals also showed that 37.8 to 68.2% of health care professionals had occupational stress [30, 31].

Studies conducted in different country showed that factors like; work overload, working unit, work experience, sex, conflict at work place, marital status, educational status, job satisfaction, working environment and not being rewarded were significantly associated with occupational stress among health care professionals [14, 32–34].

Although different studies showed the prevalence of occupational stress among health care professionals, still there is very limited evidence regarding the management of occupational stress [35, 36] and in Ethiopia there are no any strategies designed to reduce this problem. Even though there are different single studies in Ethiopia, their results showed a wide range of discrepancy on the prevalence of occupational stress among health care professionals. Therefore, conducting systematic review and meta-analysis is important to provide strong evidence for policy makers and this systematic review and meta-analysis was aimed to assess the pooled prevalence of occupational stress and its associated factors among health care professionals in Ethiopia.

## Research questions

1. What is the pooled prevalence of occupational stress among health care professionals in Ethiopia?
2. What are associated factors of occupational stress among health care professionals in Ethiopia?

## Methods

### Identification of studies

This systematic review and meta-analysis was conducted in accordance to the guideline of Preferred Reporting Items for Systematic reviews and Meta-Analyses (PRISMA) [37]. In this review, we explored PubMed, Hinari, and Science direct databases. Goggle scholar and Google searches were also done for grey literatures. In addition, the reference lists of published articles were scrutinized to pinpoint other important articles which didn’t accessed in database searches. We started searching of primary articles in June 16/2020 and continued until July 25/2020. We used English language for searching. For objective one, the keywords used were Prevalence AND work related stress OR job related stress OR occupational stress AND health care professionals OR nurse OR doctor OR psychiatrist OR pharmacist OR midwifery AND Ethiopia. We used determinants OR factors OR predictors AND occupational stress OR work related stress AND health care professional’s OR nurse OR doctor OR psychiatrist AND Ethiopia to identify associated factors for occupational stress (objective two).

### Eligibility criteria of the articles

We have used a prepared eligibility assessment format to select articles to be included in this systematic review and meta-analysis as stated below.

### Inclusion criteria

#### Study settings

Studies conducted across different regions of the country (Ethiopia) has been considered.

#### Study design

Observational studies including cross-sectional, case-control and cohort studies with original data reporting at least the prevalence or/and its associated factors of occupational stress among health care professionals were considered.

#### Language

In this review, we included articles published in English language or have English language translation.

#### Publication status

Both published and unpublished articles were included in this review.

#### Study population

Articles conducted among adult health care professionals in Ethiopian (equal to or age greater than 18 years) were considered.

#### Study period

Papers available online since June 16/2020 of July 25/2020 were considered.

### Data extraction

Authors (BG and JN) individually extracted all important data using an excel spreadsheet data extraction format. Data extraction format had components like; author name, publication year, population, study design, sample size, response rate, tool and prevalence of occupational stress. Authors used two by two tables to excerpt data for objective two (factors associated with occupational stress), but the factor working unit was not used for analysis because different categories were used [30, 38–40] which was challenging to extract data. So, after extensive communication between the data extractors we decided to remove working unit from meta-analysis. Any other differences during data extraction time between the two authors (BG and JN) were managed through communication with third and the fourth authors.

### Outcome measurement

In this systematic review and meta-analysis, we addressed two objectives. The primary objective was to assess the pooled prevalence of occupational stress among health care professionals in Ethiopia. Occupational stress is harmful physical and emotional responses that occur when the requirements of the job do not match the capabilities, resources and needs of the worker [1]. The prevalence of occupational stress was estimated by dividing the number of health professionals with stress to the total number of health professionals included in the study and multiplied by 100. The next objective of this review was to determine the pooled effects of factors on occupational stress among health professionals in Ethiopia. To evince the pooled effects, odd ratio (OR) calculated from 2 × 2 table was used.

### Quality assessment of included studies

The Ottawa Newcastle Scale adapted for cross sectional study was used to evaluate the qualities of included studies [41]. Using this tool, BG and JN evaluated the studies individually. The tool had three main parts; the first part used to measure the methodological quality of the study, the second part is used to examine the comparability of the study and the last section measures the quality of the studies with respect to statistical method appropriateness. In this review, the quality of each study was assessed and those with high quality (scored 6 and above out of 10) were incorporated for analysis. In the course of this review and meta-analysis, any variations were resolved through discussion and by using the average result of evaluators.

### Statistical procedures

Microsoft excel format was used to extract important data from each study and the extracted data were exported to STATA software version 13 for analysis. Binomial distribution formula was used to calculate the standard error of prevalence for each original article. To check the heterogeneity of the included studies, we conducted I^2^ test (a measure of the proportion of total variability explained by heterogeneity rather than chance expressed as a percentage) and affirmed as low, moderate, and high heterogeneity if it is < 50, 50–75% and > 75% respectively [42]. Leave one study out sensitivity analysis was conducted to explore the degree to which the main finding of a systematic review is affected by changes in individual studies [43] by removing one study from the groups and we analyzed the remaining studies to show if there is typical difference in pooled prevalence of study after excluding single study in each group. Furthermore, subgroup analysis was also conducted based on the region where the study was conducted and publication year to identify the source of the random variations between the point estimates of the primary articles. Regarding the publication year in our countries context we observed slight change of health care system in Ethiopia after 2018 and the numbers of mental health professionals and the stress management services are also increasing from time to time. Therefore, we conducted subgroup analysis by publication year considering before and after 2017. To check publication bias funnel plot [44] and Egger’s statistical test [45] were conducted. We used a *p*-value < 0.05 to declare the statistical significance of publication bias.

## Result

### Search results

**Fig. 1:**
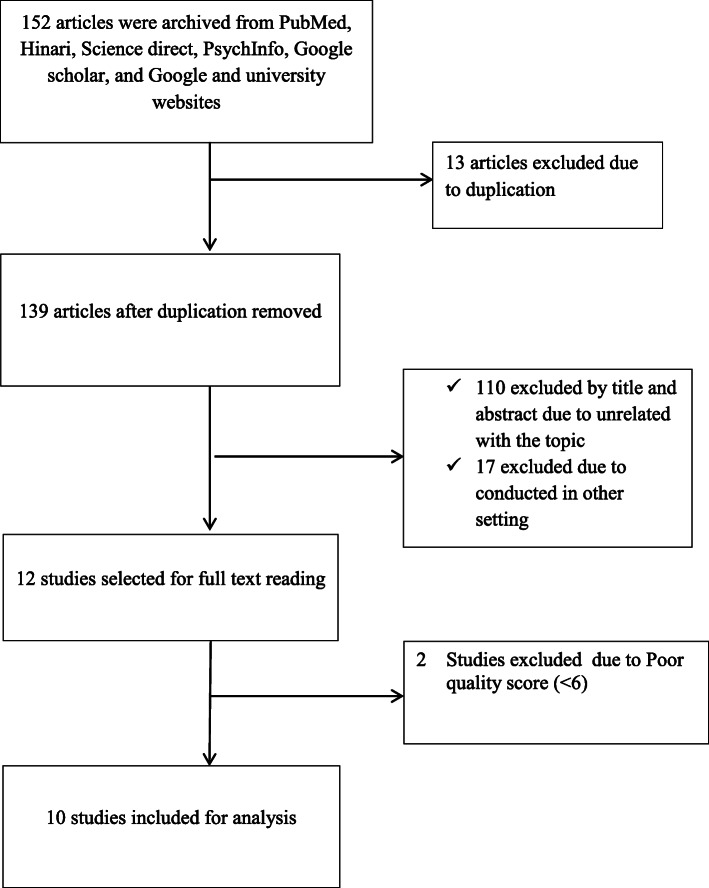
Flowchart of selected articles included in this systematic review and meta-analysis, Ethiopia, 2020

At the beginning of our search, 152 primary articles were salvaged regarding to the prevalence of occupational stress among health care professionals using PubMed, Science direct, PsychInfo, Hinari, Google and Google scholar searching engines. From the total of 152 articles, 13 articles were exempted due to duplication. Furthermore, 127 primary articles were excluded after reviewing their titles and abstracts in which we found them as unrelated to our review and conducted in other setting. Of this, we read the full texts of 12 studies and evaluated them based on our eligibility criteria. Two studies conducted in Ethiopia were also excluded due to poor quality, since the Newcastle Ottawa Scale score less than 6 [46] [Fig. 1].

### The characteristics of included articles

As described in Table 1, 10 (10) primary studies were included in this systematic review and meta-analysis. All of the included studies accompanied a total of 3137 Ethiopian health care professionals with a response rate ranging from 86.1% [47] to 98.7% [30]. All of the included primary studies were cross sectional studies and their sample sizes ranges from 138 [48] to 592 [32]. Out of the 10 included primary studies, three were from Amhara regional state [31, 47, 49], two from Addis Ababa city administration [30, 40], two from Oromia regional state [39, 48] and three were from Tigray regional state [32], Harari [38] and South Nations Nationality and peoples (SNNP) [50] (one in each). Regarding assessment tool majorities of studies used expanded nursing stress scale [38, 39, 48–50], two studies were conducted by using occupational stress scale [32, 47], another two studies nursing stress scale [30, 48] and single study was conducted by perceived stress scale which was modified to assess stress at workplace rather than stress at home [31].

**Table 1:** Distribution of studies included in this systematic review and meta-analysis, 2020

Region	Author	Publication year	Design	Sample size	Population	Response rate	Quality score	Prevalence rate	Tool
Tigray	Tafesse et al. [32]	2018	Cross sectional	592	All health care professionals	94.4	9	46.9	OCS
Amhara	Haile et al. [40]	2017	Cross sectional	181	Nurse	98.3	7	57.3	ENSS
	Zeleke et al. [38]	2019	Cross sectional	294	All health care professionals	86.1	8	48.6	OCS
	Birhanu et al. [31]	2018	Cross sectional	208	All health care professionals	95	7	68.2	Modified PSS
Oromia	Worku et al. [39]	2020	Cross sectional	405	Nurse	94	7	53	ENSS
	Nemera et al. [42]	2018	Mixed	180	Nurse	98.3	6	49.2	ENSS
Addis Ababa City	Zewdu et al. [30]	2014	Cross sectional	343	Nurse	93	7	37.8	NSS
	Tekletsadik et al. [39]	2020	Cross sectional	398	All health care professionals	98.7	8	46.8	NSS
SNNP	Anand et al. [44]	2018	Cross sectional	138	Nurse	97.8	6	56.3	ENSS
Harari	Baye et al. [43]	2015	Cross sectional	398	Nurse	92.2	6	62.2	ENSS

**Note:** ***ENSS*** Expanded nursing stress scale**,** ***NSS*** Nursing stress scale**,** ***OCS*** Occupational stress scale**,** ***PSS*** perceived stress scale

### Meta-analysis

**Fig. 2:**
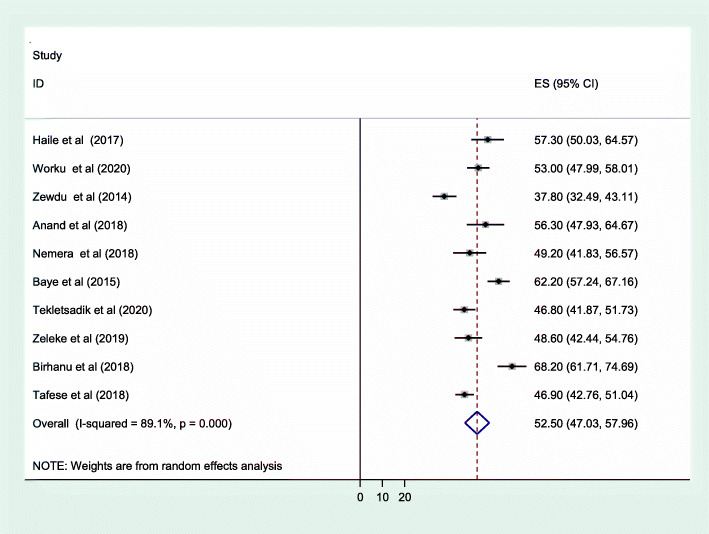
Forest plot of the included studies to determine the pooled prevalence of occupational stress among health care professionals in Ethiopia, 2020

**Fig. 3:**
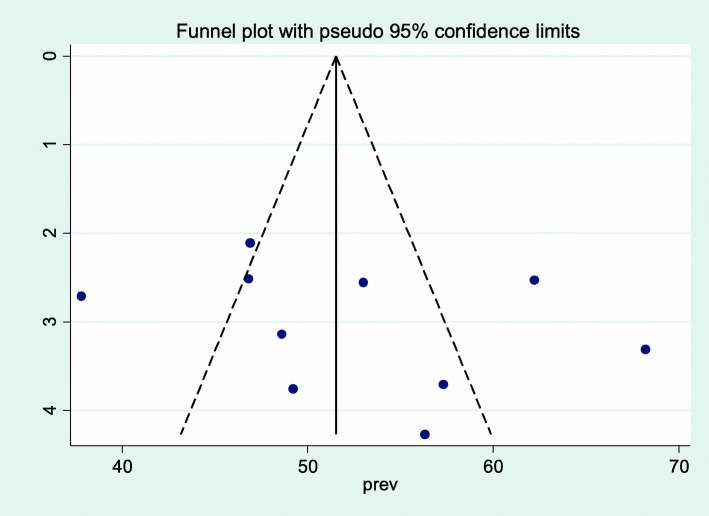
Funnel plot to assess publication bias among included studies, 2020

As shown in Fig. 2, the forest plot was conducted to show the result of the included studies. The pooled prevalence of occupational stress among health professionals in Ethiopia was 52.5 [95% CI: (47.03, 57.96)]. With regard to heterogeneity, I^2^ was conducted and there was high heterogeneity as evidenced by I^2^ = 89.1% and *p* < 0.001. Therefore, random effect model was used to estimate the pooled prevalence of occupational stress among health care professionals in Ethiopia. To detect publication bias, funnel plot was conducted as shown on Fig. 3. In addition, the objective statistical tests, Begg’s and Eggers’ tests were done and there was no evidence for publication bias (*P* = 0.283 and *P* = 0.369) respectively.

### Sensitivity and subgroup analysis

The result showed a pooled prevalence ranges between 50.75 [95% CI: (45.8, 55.69)] and 54.15 [95% CI: (49.16, 59.14)] [Table 2]. A subgroup analysis was also conducted to address whether the summary effects varies in relation to specific characteristics of the studies. The result of subgroup analysis based on the region where the studies conducted showed that health care professionals in Addis Ababa city administration had lowest prevalence of occupational stress; 42.36% [95% CI: (33.54, 51.18)] whereas, the highest prevalence was shown in Harari; 62.2% [95% CI (57.24, 67.16)] [Table 3]. We also conducted subgroup analysis by publication year. Subgroup analysis by publication year showed approximately similar prevalence of occupational stress among studies conducted in 2017 and before, and after 2017; 52.4 [95% CI; (36.52, 68.27)] and 52.49 [95% CI: (47.1, 57.87)] respectively. But the heterogeneity of studies conducted in 2017 and before was high (I^2^ = 95.6%, *p* < 0.001) as compared to studies conducted after 2017 (I^2^ = 83.6%, *p* < 0.001) [Table 3].

**Table 2:** Sensitivity analysis of the prevalence of occupational stress among health professionals in Ethiopia through leave-one study-out technique, 2020

Author name and publication year	Pooled prevalence with 95% CI	Heterogeneity	
		I^**2**^ (%)	***P*** value
Haile et al., 2017 [40]	52.00 [46.12, 57.88]	90.0	*P* < 0.01
Worku et al., 2020 [39]	52.46 [46.26 58.66]	90.2	*P* < 0.01
Zewdu et al., 2014 [30]	54.15 [49.16, 59.14]	85.1	*P* < 0.01
Anand et al., 2018 [44]	52.12 [46.26, 57.99]	90.1	*P* < 0.01
Nemera et al., 2018 [42]	52.85 [46.90, 58.81]	90.2	*P* < 0.01
Baye et al., 2015 [43]	51.34 [45.93, 56.74]	87.1	*P* < 0.01
Tekletsadik et al., 2020 [39]	53.17 [47.10, 59.24]	89.8	*P* < 0.01
Zeleke et al., 2019 [38]	52.94 [46.91, 58.97]	90.2	*P* < 0.01
Birhanu et al., 2018 [31]	50.75 [45.81, 55.69]	85.4	*P* < 0.01
Tafesse et al., 2018 [32]	53.18 [47.02, 59.34]	89.5	*P* < 0.01

**Table 3:** Subgroup prevalence of occupational stress among health care professionals in Ethiopia, 2020 (*n* = 10)

Variables	Characteristics	No. of studies	Prevalence (95% CI)	Heterogeneity	
				I^**2**^ (%)	***P***-value
Region and city Administration	Amhara	3	58.01 [46.39, 69.62]	89.1%	*P* < 0.01
	Oromia	2	51.80 [47.65, 55.94]	0%	*P* = 0.40
	Addis Ababa city	2	42.36 [33.54, 51.18]	83.1%	*P* = 0.01
	SNNP	1	56.30 [47.93, 64.67]	–	–
	Tigray	1	46.90 [42.76, 51.04]	–	–
	Harari	1	62.20 [57.24, 64.67]	–	–
Publication year	2017 and before	3	52.40 [36.52, 68.27]	95.6	*P* < 0.01
	After 2017	**7**	52.50 [47.03, 57.96]	83.6	*P* < 0.01

### Factors associated with occupational stress

In the included primary studies, a lot of factors were identified as predictors for occupational stress. But in this systematic review and meta-analysis, factors that were recorded in at least three primary articles were taken for meta-analysis. We identified sex [30, 32, 48, 49], marital status [30, 32, 39, 48], work experience [32, 39, 47, 49] and working unit as factors for occupational stress among health care professionals in Ethiopia.

**Fig. 4:**
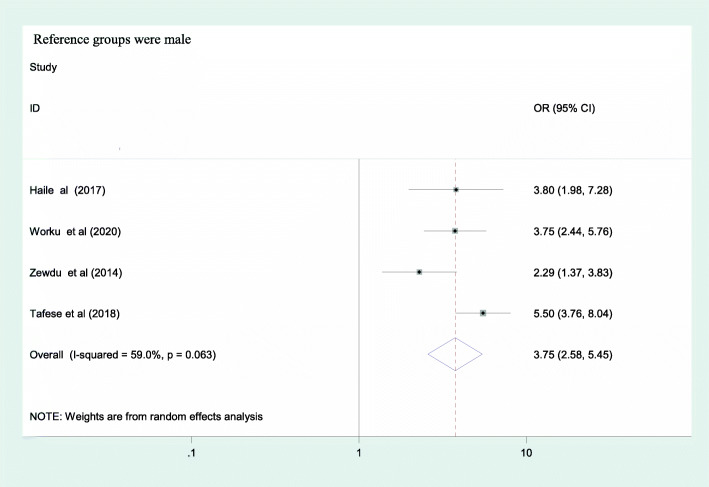
Association between sex and occupational stress among health care professionals in Ethiopia, 2020

**Fig. 5:**
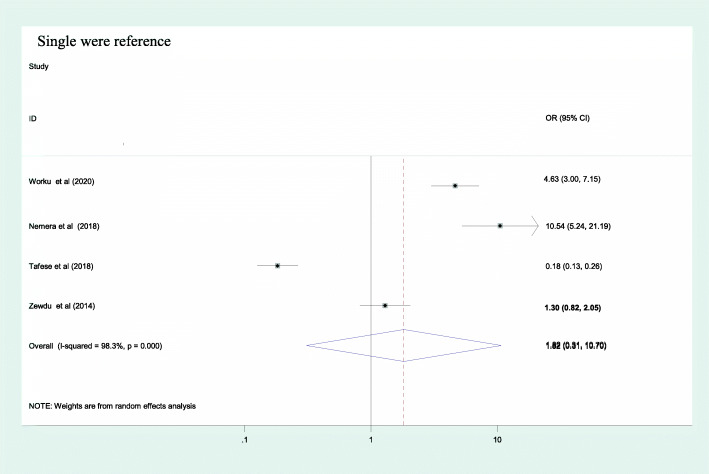
Association between marital status and occupational stress among health care professionals in Ethiopia, 2020

**Fig. 6:**
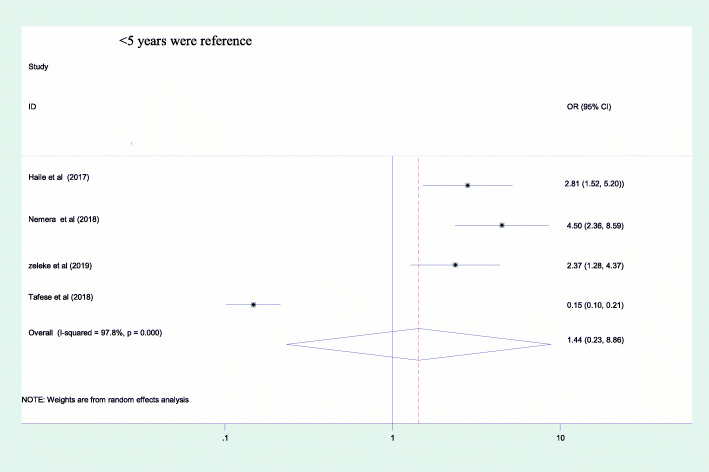
Association between work experience and occupational stress among health care professionals in Ethiopia, 2020

From the included factors, only sex of the health care professionals was identified as a predictor for occupational stress as evidenced by a pooled effect of 3.75 [95%CI: (2.58, 5.45)] [Fig. 4]. Female participants had 3.7 times greater odds of having occupational stress as compared to their counterparts. The other two factors; marital status and working experience had no association with occupational stress as evidenced by their confidence interval including 1; 1.82 [95% CI: (0.31, 10.7)] and 1.43 [95% CI: (0.23, 8.86)] respectively [Figs. 5 & 6].

## Discussion

In this review and meta-analysis, the pooled prevalence of occupational stress was 52.5 [95% CI: (47.03, 57.96)]. This finding was almost comparable with studies done in Portugal (50%) [51], South Africa (51%) [52] and Iran (49.5%) [53]. However, it was high as compared to studies done in Saudi Arabia (45.5%) [54], Jordan (30%) [55] and Australia (41.2%) [56]. Whereas, it was low as compared to studies done in India (87.4%) [9], Iran (78.4%) [57], Saudi Arabia (66.2%) [26] and United Kingdom (59%) [58].

The findings of different studies showed that the prevalence of occupational stress among health care professionals varies by countries, tools and socioeconomic levels. It is important to consider that this research is based on the results of 10 studies, while the researches in other countries are based different numbers of articles. To explain the possible reasons for the variations of findings within countries, the first reason might be due to discrepancy in sample size and study population. For instance, in studies conducted at India and Iran the study participants were only nurses and the general population were used in Australia.

The other possible reason for the difference might be due to variations in socioeconomic level and measurement tool; in Saudi [54] and Jordan [55] occupational stress scale [55] which had some variation in numbers of components as compared to nursing stress scales [59] that was used by most of studies included in this review.

Regarding the factors associated with occupation stress among health care professionals, being female had higher odds of developing occupational stress; OR: 3.75 [95% CI: (2.58, 5.45)] and was similar with studies done in Iran [60], United Kingdom [58] and Japan [61]. This might be because of female had multiple role outside their work place. However, study done in Nigeria showed no difference [29]. The reason could be as a result of difference in socio-cultural and study population.

The first limitation of this study might be due to the cross sectional nature of the included studies which couldn’t show the temporal relationship between the outcome and independent variables. The second, un-proportional distribution of study subjects (nurses number were high). The last limitation might be due to presence of heterogeneity between studies, as result readers should consider during using this finding.

## Conclusion

The finding of this review and meta-analysis showed that half of health professionals in Ethiopia experienced occupational stress. Being female participant was significant predictor for occupational stress. Therefore, introduction of policies supporting health care professionals well-being at work in Ethiopia are advisable, in addition to the analysis of its impact on the health care system. Although the analysis of the effects of occupational stress among health care providers was not analyzed, this review underlined that this global problem warrants further research in developing country.

## Data Availability

The data included in this study is available and can be accessed by contacting the corresponding author through this email address; bekahegngi@gmail.com or Bekahegng@du.edu.et.

## Abbreviations

CI: Confidence interval
OR: Odd ratio
SNNP: South Nations and Nationalities People
